# A Mendelian randomization study of the associations between depression, anxiety and clinical conditions including thyroid nodules, flatulence, and irregular menstruation

**DOI:** 10.1097/MD.0000000000044041

**Published:** 2025-08-22

**Authors:** Zheng Bi, Lujie Wang, Jinju Li, Jiawen Jing, Zhaohui Fang

**Affiliations:** a Department of Endocrinology, The First Affiliated Hospital of Anhui University of Traditional Chinese Medicine, Hefei, Anhui, People’s Republic of China; b Graduate School Anhui University of Chinese Medicine, Hefei, Anhui, People’s Republic of China.

**Keywords:** anxiety, depression, irregular menstruation, Mendelian randomization, physical health, thyroid nodules

## Abstract

**Objective:**

Given the high prevalence of psychological and physical conditions as well as the limited understanding of the potential causal links, we aim to explore the associations between psychological problems (depression and anxiety) and physical health issues (thyroid nodules, flatulence, menstrual disorders).

**Methods:**

Genetic data on depression and anxiety were sourced from the Psychiatric Genomics Consortium and the FinnGen database, respectively. Meanwhile, datasets of the Epidemiological Network were employed to investigate thyroid nodules, flatulence, and menstrual irregularities. The Mendelian randomization (MR) analysis was conducted with the TwoSampleMR R package. To ensure the accuracy and detect horizontal pleiotropy, the inverse variance weighting, MR-Egger regression, and weighted averaging were employed. Additionally, the Cochran *Q* heterogeneity test was applied to assess the heterogeneity. Moreover, the stability of the findings was assessed using the leave-one-out method. Finally, the supplementary MR and multivariate MR analyses were utilized to verify the consistency of the associations.

**Results:**

Our analysis demonstrated that depression significantly influenced the development of thyroid nodules (*P* = .034, odds ratio = 1.75, confidence interval [1.0419, 2.9392]) and irregular menstruation (*P* = .02, odds ratio = 1.005, confidence interval [1.0008, 1.0097]) but had no effect on flatulence (*P* = .156). However, the inverse variance weighting *P*-values for anxiety in all outcomes showed no causal association with any of the studied conditions.

**Conclusions:**

MR analysis showed that depression causally worsens thyroid nodules and menstrual irregularities, while anxiety has no such association. Moreover, depression continuously impacts menstrual irregularities, even after anxiety adjustment. Our findings highlight the importance of mental health in managing physical health.

## 1. Introduction

In recent years, as the relationship between mental and physical health has received increasing attention in medical research, many physical conditions have been associated with depression and anxiety, highlighting the interaction between the mind and body.^[1,2]^ Depression is an important factor in global health and is associated with several diseases such as heart disease, blood diabetes, and endocrine abnormalities including thyroid disorders.^[3–5]^ A 7-year (2011–2018) clinical follow-up study demonstrated that participants with chronic diseases at baseline had a higher risk of depression. This risk increased significantly with the number of chronic conditions, and by the end of the follow-up, 41.2% of the participants had developed depression.^[6]^ Anxiety disorders, which affect the majority of the population, are linked to gastrointestinal disorders, such as irritable bowel syndrome, gastroesophageal reflux disease, and reproductive disorders such as menstrual irregularities.^[7,8]^ Specifically, researchers have pointed out that the prevalence of thyroid nodules in the Chinese general population is approximately 36.9%, with higher detection rates in women and older individuals.^[9]^ Additionally, patients with thyroid nodules often experience higher levels of anxiety and depression.^[10]^ Likewise, Catherine found that Severe flatulence is associated with somatization, and most of the patients suffering from it are young females.^[11]^ Moreover, data from a systematic review indicates that depression and anxiety affect 9.6% to 69.3% of women of childbearing age, and most of these women experience irregular menstruation.^[12–14]^ Despite the fact that the causal pathways connecting depression and anxiety to these conditions are not yet clear, it is believed that they involve both direct biological processes and indirect behavioral influences.

Mendelian randomization (MR) has become an indispensable epidemiological methodology.^[15,16]^ By leveraging genetic variants randomly assigned at conception as instrumental variables, it effectively addresses the confounding issue that has long plagued traditional observational studies.^[17]^ This unique approach allows MR to establish more robust causal relationships between genetic factors and diseases, thereby enhancing the reliability and validity of research findings.^[18]^ This study used the method to explore the connection between depression/anxiety and thyroid nodules, flatulence and irregular menstruation. The rationale for targeting these specific conditions rests on 2 essential elements: existing epidemiological evidence and the possible overlap between condition-related genes and relevant biological pathways.^[19]^

In this study, we used the Psychiatric Genomics Consortium (PGC) genetic data on stress, with a focus on depression, and evaluated the associated health outcomes using data from the integrative epidemiology unit (IEU) FinnGen database. To confirm the results and eliminate pleiotropic effects, we applied different MR techniques such as IVW and MR-Egger.^[20]^ This study aim to investigate the genetic link between depression and anxiety and the impact on physical health, which is crucial for developing treatment strategies that simultaneously address mental and physical health, thus improving patient outcomes.

## 2. Material and methods

### 2.1. Data sources and selection

#### 2.1.1. Exposure data

Depression: Exposure data for depression were sourced from the PGC database (https://pgc.unc.edu/). The data were extracted from a comprehensive study by Wray et al (2018). The dataset comprised a substantial sample of 4,80,359 participants, including 1,35,458 diagnosed cases of depression and 3,44,901 controls without the condition. Anxiety: for exposure to anxiety, data were sourced from the FinnGen database, specifically from Release R10. This database, renowned for its major contributions to genetic research, provides a comprehensive summary dataset named KRA_PSY_ANXIETY_EXMORE. The dataset comprised 3,46,542 participants, including 44,663 cases of anxiety disorders and 3,01,879 controls. In addition, the Beck Depression Inventory was utilized for assessing depression severity and the generalized anxiety disorder 7-item scale was employed for evaluating anxiety symptoms.^[21,22]^

#### 2.1.2. Outcome data

Thyroid nodule: The thyroid nodule data were meticulously extracted from the IEU database, specifically under the identifier finn-b-E4_GOITRENOD. This dataset included 1,88,805 individuals comprising 1121 diagnosed cases and 1,87,684 control participants. Flatulence: for the outcome of flatulence, data were sourced from the IEU database, specifically under the identifier ukb-a-584. This dataset included 3,37,199 individuals, with 201 identified as cases of flatulence and 3,36,998 serving as controls. Irregular menstruation: data concerning irregular menstruation were also derived from the IEU database, specifically under ID ukb-a-578. The dataset consisted of a large sample of 3,37,199 individuals, with 6882 cases of irregular menstruation and 3,30,317 controls.

#### 2.1.3. Data filtering

In the forward MR approach, single nucleotide polymorphisms (SNPs) was selected to satisfy stringent criteria: a *P*-value below 5e−8 was chosen following common genome-wide association study practice to control false positives from multiple testing, ensuring strong genetic associations with the exposure. Notably, we implemented a multi-tiered filtering approach for SNP selection. Specifically, SNPs were retained only if they met the following stringent criteria: minor allele frequency (MAF) ≥ 1%, linkage disequilibrium coefficient below 0.001 within a genomic window of 10 Mb, exclusion of intermediate-frequency palindromic SNPs (A/T or C/G), SNPs demonstrating significant association with study outcomes (*P* < 5 × 10^−8^), and those with missing genotype data exceeding 5%. For non-palindromic SNPs, strand orientation was verified by comparing MAF between exposure and outcome datasets, with realignment performed when absolute MAF difference ≤ 0.3. In the reverse analysis, acknowledging the limited number of available SNPs, a slightly more lenient threshold (*P* < 5 × 10^−6^) was adopted to maintain statistical power. Additionally, the linkage disequilibrium parameter was adjusted to *r*^2^ = 0.01 to accommodate the dataset’s analytical requirements.

## 3. MR analysis

### 3.1. MR methodology

The R package of twoSampleMR package (Version 0.5.10; https://mrcieu.github.io/TwoSampleMR/index.html) was used to perform MR analysis with depression and anxiety as exposure and thyroid nodules, flatulence, and irregular menstruation. IVW: this method provides estimates of individual SNP values using a weighting technique, where each SNP’s predicted probability is weighted by the inverse of its difference. MR-Egger: addresses potential pleiotropy through interference in regression models, allowing for direct influence of SNPs on the outcome. Weighted median: this assumption assumes that more than half of the total weighting is from valid instruments. Simple type: estimate the type of individual SNP effect. Type stress: a powerful predictor that adds richness to reduce the impact of outlying SNPs.

### 3.2. Heterogeneity assessment

Heterogeneity testing was carried out using 2 methods, namely MR-Egger and inverse variance weighted. Whether there was heterogeneity was determined based on the *Q* value obtained from these 2 methods. When *P* > .05, it was indicated that there was no heterogeneity. The effectiveness of the selected instrumental variables was reflected by assessing the differences in the results obtained from different instrumental variables. Specifically, the mr_heterogeneity function in TwoSampleMR was utilized to calculate Cochran’s *Q* statistic, that is, *Q* = ∑_*j*_[*w*_*j*_ (β ^_*j*_−β)^2^].

Here, β ^_*j*_ represents the coefficient estimate obtained from the *j*th instrumental variable, *w*_*j*_ is the corresponding weight, and β is the pooled estimate obtained through combination using either the IVW or the MR-Egger method.

### 3.3. Pleiotropy test

The MR-Egger regression was employed to test for pleiotropy. In detail, when the Egger intercept significantly deviated from 0, it indicated the presence of horizontal pleiotropy. In addition, Mendelian Randomization Pleiotropy RESidual Sum and Outlier was also utilized for the pleiotropy test, and the *P*-value was calculated based on 1000 simulations. An indication that *P* > .01 suggested that there was no horizontal pleiotropy.^[23]^

### 3.4. Multivariable MR (MVMR)

After adjusting for the mutual influence between depression and anxiety, a multivariable Mendelian randomization (MVMR) analysis was conducted. The IVW method was used to evaluate the causal effects of depression and anxiety on thyroid nodule, flatulence, and irregular menstruation, respectively. For the MVMR, the following points should be noted: Generally speaking, for a SNP, it only needs to be strongly correlated with one of the exposures (*P* < 5e−8, LD *r*^2^ < 0.001); Information of SNPs in all exposures and outcomes should be extracted, and there should be no missing data; Complete genome-wide association study results regarding the exposures and outcomes are required for conducting the MVMR analysis.

## 4. Results

### 4.1. Forward MR data filtering

To ensure the quality of genetic instrumental variables and improve the reliability of the data, Forward MR Data Filtering was carried out. For the depression-related analysis, 59 SNPs were initially selected following a strict genetic association criteria. Subsequently, to align the SNPs with the outcome data, the “harmonise_data” function was employed. Following this, SNPs that failed to meet the mr_keep = FALSE criterion were excluded. As a result, the final counts of SNPs suitable for MR analysis were 59 for thyroid nodules, 55 for flatulence, and 55 for irregular menstruation, indicating a tailored approach to each health outcome based on genetic data quality and relevance. Similarly, for anxiety, the MR data filtering was performed with the same number of 52 SNPs. After undergoing a similar process of exclusion for SNPs that were flagged as “mr_keep = FALSE”, the final total comprised 13 SNPs for each outcome. The detailed information of forward MR data filtering was illustrated in Table S1, Supplemental Digital Content, https://links.lww.com/MD/P726.

### 4.2. Reverse MR data filtering

To explore potential reverse causal relationships, specific SNPs were selected as genetic instruments. Specifically, for thyroid nodules, flatulence, and irregular menses, the initial SNP counts were 6, 44, and 13, respectively. After that, they were aligned with the exposure data for depression and anxiety to establish associations between genetic variations and potential outcomes. In detail, for depression, the final counts were 5, 42, and 12 for thyroid nodules, flatulence, and irregular menstruation, respectively. For anxiety, the final SNP counts were 6, 44, and 13 for the same conditions, indicating strict filtering and data refinement. The detailed information of reverse MR data filtering was illustrated in Table S2, Supplemental Digital content, https://links.lww.com/MD/P727.

### 4.3. Forward MR outcomes

**Figure 1.**
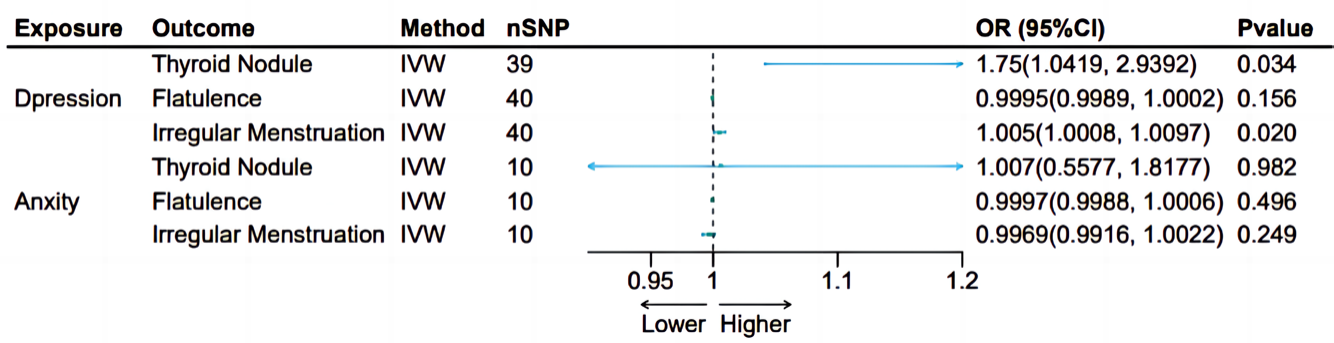
Forest plot of odds ratios from Mendelian randomization analyses. nSNP used in Mendelian randomization analyses; OR > 1 indicates that the exposure is associated with an increased risk of the outcome, whereas OR < 1 indicates a decreased risk; results with *P*-value < .05 were considered statistically significant. CI = confidence interval, nSNP = number of single nucleotide polymorphism, OR = odds ratio, IVW = inverse variance weighting.

**Figure 2.**
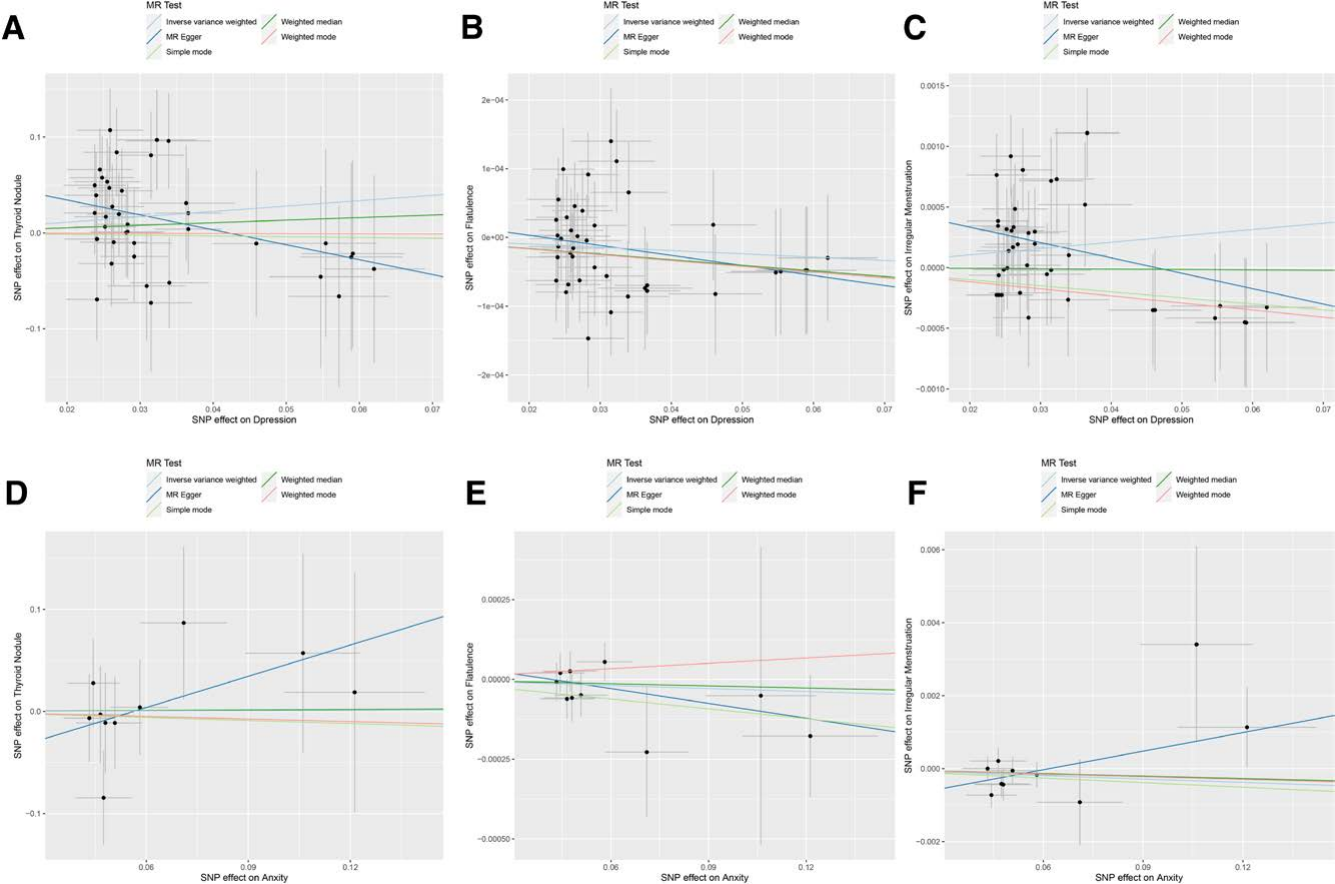
Scatter plots of forward MR analyses showing associations between exposures and outcomes. (A–C) Scatter plots for the association of depression with thyroid nodules, flatulence, and irregular menstruation, respectively. (D–F) Scatter plots for the association of anxiety with thyroid nodules, flatulence, and irregular menstruation, respectively. A slope > 0 indicates a positive association (i.e., the exposure is associated with an increased risk of the outcome), while a slope < 0 indicates a negative association (i.e., the exposure is associated with a decreased risk of the outcome). All plots are based on results from the IVW method. IVW = inverse variance weighting, MR = Mendelian randomization.

**Figure 3.**
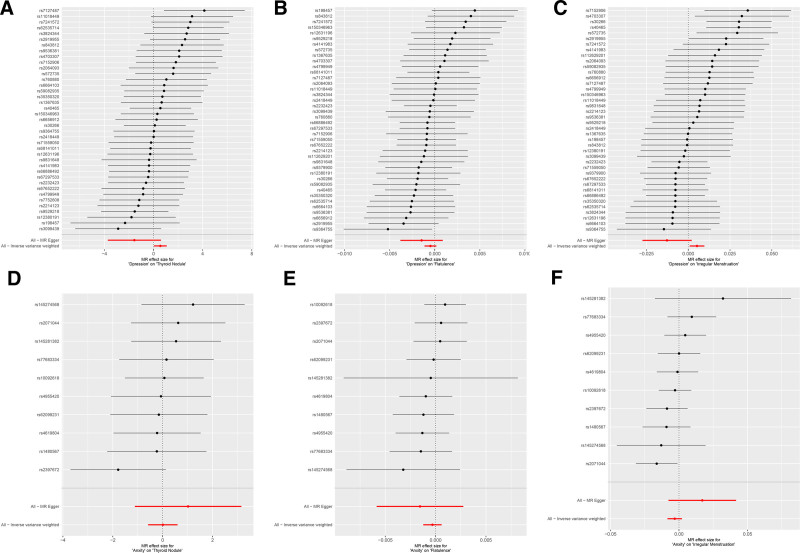
Forest plots of forward MR analysis results. (A–C) Forest plots showing MR effect sizes of depression on thyroid nodules, flatulence, and irregular menstruation. (D–F) Forest plots showing MR effect sizes of anxiety on thyroid nodules, flatulence, and irregular menstruation. Each point represents the effect size of an individual SNP on the outcome, with horizontal lines indicating 95% CIs. Values > 0 indicate a promoting effect on the outcome, while values < 0 indicate an inhibitory effect. The red line denotes the combined effect size estimated by the IVW method, which was used as the primary analytical approach. IVW = inverse variance weighting, MR = Mendelian randomization, SNP = single nucleotide polymorphism.

The results of forward MR analysis are shown in Figure 1, which provide a significant insight into the impact of depression on specific physical health outcomes. Figures 2 and 3 are a scatter plot and a forest plot of the result respectively. Overall, these evaluations confirmed a statistically significant causal connection, demonstrating a notable exacerbation in the occurrence of thyroid nodules due to depression, with an IVW *P*-value of .034 and an odds ratio (OR) of 1.75, encapsulated within a 95% CI of [1.0419, 2.9392] (Figs. 2A and 3A). Similarly, a substantial causal link was found between depression and the prevalence of irregular menstruation, where the IVW_*P* value was even lower at 0.02, and the OR was 1.005, within a tightly bound 95% CI of [1.0008, 1.0097], indicating a discernible yet quantifiable effect (Figs. 2C and 3C). By contrast, the results did not indicate a causal relationship when examining the potential influence of depression on flatulence. The IVW_ *P* value for this analysis was .156, signifying that depression did not appear to influence flatulence events in any significant way according to the genetic tools used in this study (Figs. 2B and 3B).

Regarding the effects of Anxiety, the MR outcomes were quite different. The analyses for anxiety showed no significant causal relationship with any of the following conditions: thyroid nodules, flatulence, or irregular menstruation. The respective IVW_*P* values for these outcomes all indicated non-significance, underscoring that Anxiety, as measured through the genetic variants selected for this study, does not have a detectable causal impact on these particular physical health conditions (Fig. 2D–F).

Moreover, to ascertain the robustness and credibility of these findings, MR evaluations used both the IVW and MR-Egger methods for each SNP involved in the study. These detailed analyses, depicted in Figure 3, further substantiate the results by providing a comprehensive view of the individual SNP effects and their contribution to the overall causal estimates. This study shows the difference in the relationship between mental disorders, such as depression, and their impact on physical health, underscored by the evaluation of genetic causality. The differences in the relationship between different situations and mental health emphasizes the need for a clear understanding of the genetic background of such associations.

### 4.4. Sensitivity analysis

To evaluate the impact of individual SNPs on patient outcomes, sensitivity analysis systematically excluded each SNP and recalculated the meta-effects, thereby determining whether any single SNP can significantly alter the overall results and ensuring the robustness of the findings. In detail, we further explored the reliability of our MR studies using comprehensive hypothesis testing, including tests for heterogeneity and pleiotropy. These tests are essential for guaranteeing the reliability of the causal conclusions drawn from our study.

### 4.5. Heterogeneity testing

To evaluate the consistency of the individual SNP predictions used in our MR analysis, the heterogeneity tests were performed. The results of these tests, detailed in Table 1, showed no significant differences between the SNPs associated with beneficial outcomes. In particular, the tests confirmed consistency in SNP effects for depression-related conditions such as thyroid nodules and irregular menstruation. The lack of significant differences in these analyses increased the confidence of our findings, indicating that genetic tools provide a consistent assessment of the effects of depression. High health benefits for the body.

**Table 1:** Results of heterogeneity tests.

Exposure	Outcome	Method	*Q*	*Q*_df	*Q*_*P*val
Depression	Thyroid nodule	MR-Egger	30.47	37	0.77
		Inverse variance weighted	34.36	38	0.64
	Flatulence	MR-Egger	31.68	38	0.76
		Inverse variance weighted	32.41	39	0.76
	Irregular menstruation	MR-Egger	45.38	38	0.19
		Inverse variance weighted	52.74	39	0.07
Anxiety	Thyroid nodule	MR-Egger	4.67	8	0.79
		Inverse variance weighted	5.61	9	0.78
	Flatulence	MR-Egger	4.49	8	0.81
		Inverse variance weighted	4.83	9	0.85
	Irregular menstruation	MR-Egger	6.57	8	0.58
		Inverse variance weighted	9.24	9	0.42

When the *Q*_*P*val is greater than 0.05, it is considered that there is no heterogeneity, with primary reliance on the inverse variance weighted method.

MR = Mendelian randomization.

### 4.6. Pleiotropy level testing

In addition to evaluating heterogeneity, pleiotropy level tests were conducted to examine whether the SNPs used as instruments in the MR study influenced the outcomes by routes other than the main exposure, which, in this case, was depression. The results of these tests, presented in Table 2, indicate that the SNPs used to assess the impact of depression on thyroid nodules and irregular menstruation do so without any significant pleiotropic bias. This finding suggests that the identified causal pathways are uniquely related to the impact of depression and do not involve other indirect genetic effects that might distort the analysis.

**Table 2:** Results of pleiotropy level tests.

Exposure	Outcome	Egger_intercept	SE	*P* value
Depression	Thyroid nodule	0.065446	0.03	.06
	Flatulence	3.22E−05	3.77E−05	.40
	Irregular menstruation	0.000588	0.000237	.02
Anxiety	Thyroid nodule	−0.05743	0.06	.36
	Flatulence	6.44E−05	0.000112	.58
	Irregular menstruation	−0.00105	0.000642	.14

It is considered that horizontal pleiotropy does not exist when the *P*-value > .01.

SE = standard error.

The lack of significant pleiotropy reinforces the validity of the MR findings, confirming that the genetic variants implicated in depression genuinely affect the risk of developing thyroid nodules and that irregular menstruation is directly related to their association with depression. This clean bill of health in terms of pleiotropy is essential to draw reliable causal conclusions from genetic association studies.

Together, these sensitivity analyses, both the heterogeneity tests and pleiotropy level tests, provide a robust foundation for the conclusions drawn regarding the genetic foundations of the impact of depression on thyroid nodules and irregular menstruation. They ensured that the observed effects were due to the direct impact of depression mediated through the specific SNPs analyzed, rather than the underlying genetic confounders or methodological biases.

### 4.7. Leave-one-out analysis

**Figure 4.**
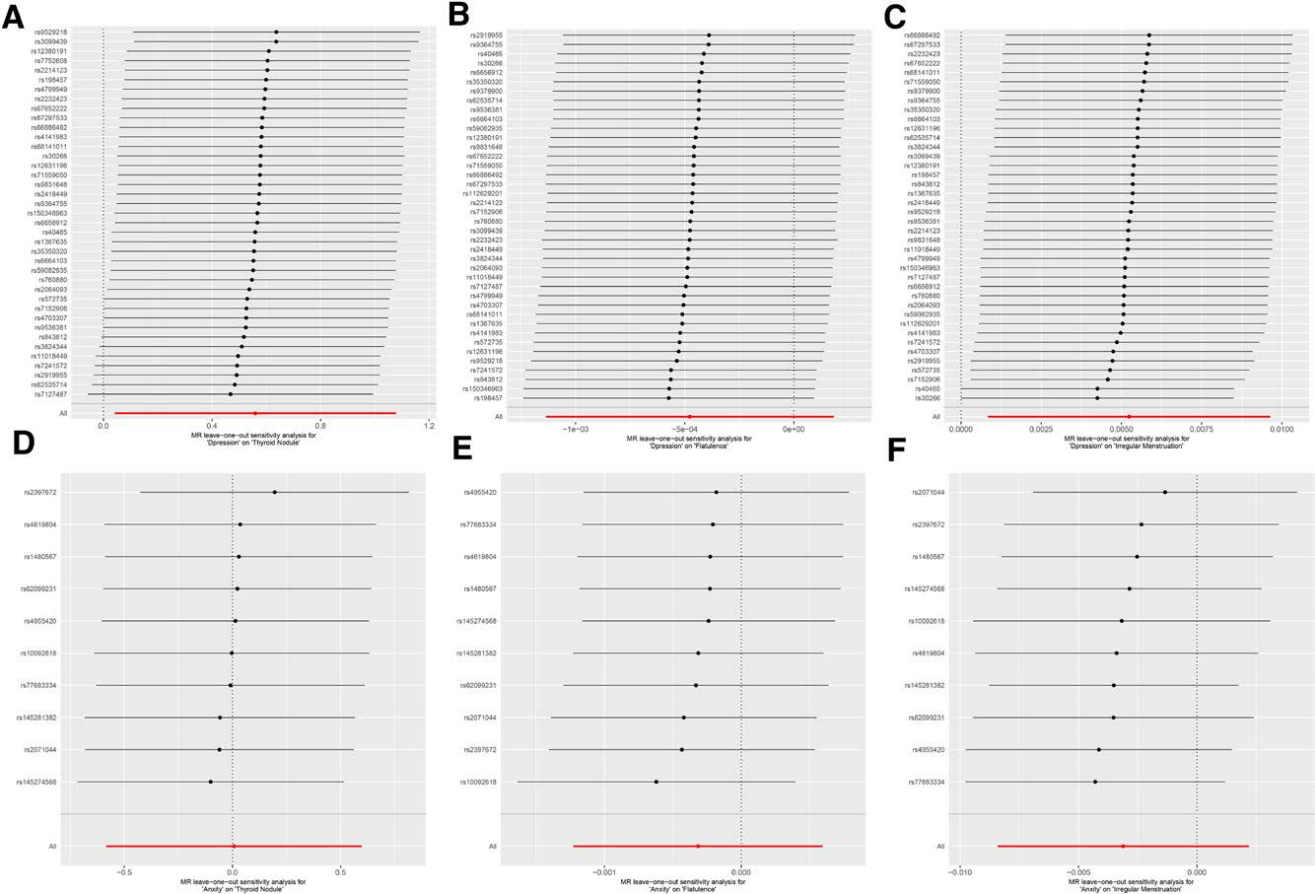
Forest plots of leave-one-out sensitivity analyses. (A–C) MR leave-one-out analyses for the effect of depression on thyroid nodules, flatulence, and irregular menstruation. (D–F) MR leave-one-out analyses for the effect of anxiety on thyroid nodules, flatulence, and irregular menstruation. Each black dot represents the causal effect estimate of the remaining SNPs after excluding 1 SNP. A stable overall effect indicates that the MR results are not driven by a single outlier SNP but reflect the collective influence of all SNPs included. MR = Mendelian randomization, SNP = single nucleotide polymorphism.

**Figure 5.**
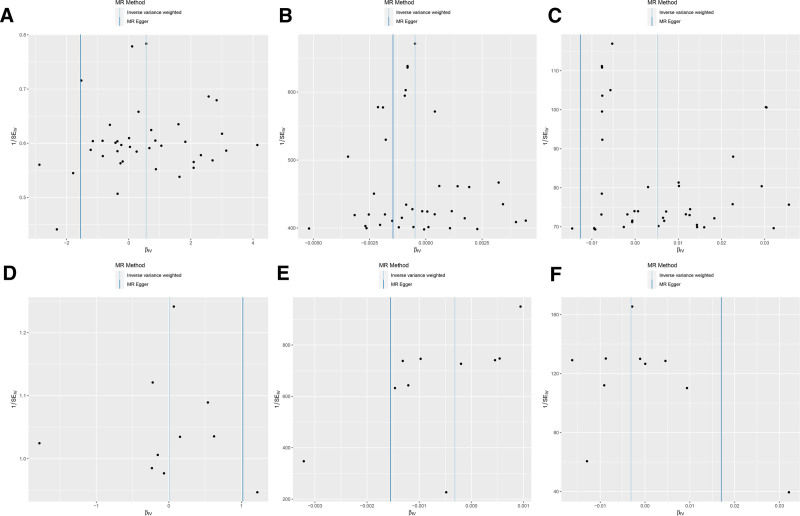
Funnel plots for assessment of publication bias. (A–C) Funnel plots showing the MR effect sizes of depression on thyroid nodules, flatulence, and irregular menstruation, respectively. (D–F) Funnel plots showing the MR effect sizes of anxiety on thyroid nodules, flatulence, and irregular menstruation, respectively. Each black dot represents an individual SNP. The vertical line indicates the pooled effect size estimated by the IVW method. Symmetric distribution of SNPs around the IVW line suggests no significant publication bias, while asymmetry may indicate potential bias or heterogeneity. MR = Mendelian randomization, SNP = single nucleotide polymorphism, IVW = inverse variance weighting.

To assess the influence of individual SNPs on the overall MR predictions, the leave-one-out analysis was conducted, the findings from this analysis, as illustrated in Figure 4. Specifically, Figure 4A–C shows that all the error bars are mostly to the right of 0 on the *x*-axis. This stability, with little change throughout the regression analysis, demonstrates the stability and reliability of our MR findings, confirming that the associations are not objects. Artificially produced by genetic mutations. In addition, as illustrated in Figure 5, the distribution of all SNPs around the regression line for both the IVW and MR-Egger methods was statistically significant, which is critical because it suggests that the causal inferences obtained from our MR analysis are well-balanced and robust, avoiding excessive reliance on any single SNP. The consistent positioning of these SNPs around the central regression lines indicates that the MR estimates are stable and reliable, reflecting a true genetic association rather than being skewed by outlier SNPs or extreme values.

### 4.8. Reverse Mendelian randomization outcomes

**Figure 6.**
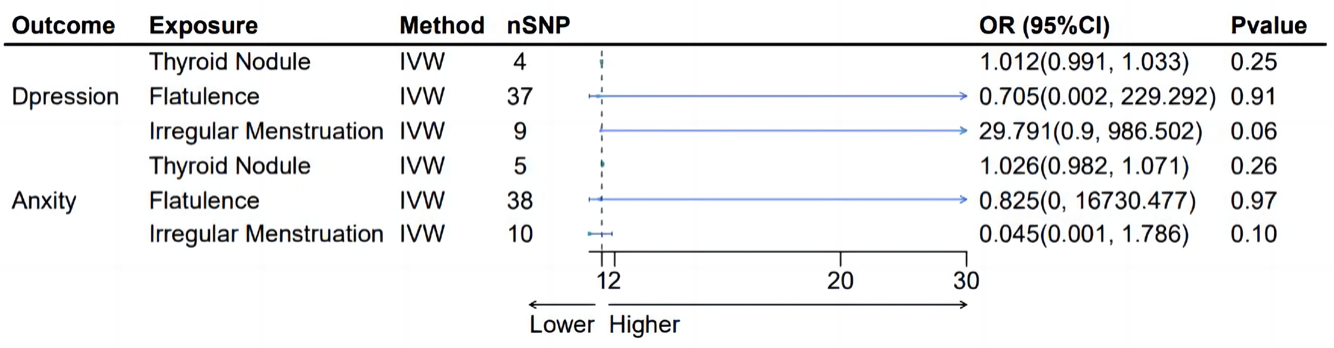
Forest plot of odds ratios from reverse MR analyses. OR > 1 indicates that the outcome may promote the occurrence of exposure, while ORs < 1 suggests a potential inhibitory effect. Statistical significance was determined using a 2-tailed test with a threshold of *P* < .05. In the present analyses, all OR had *P*-values > .05, indicating no statistically significant evidence of reverse causality, which is consistent with the study’s primary assumptions. MR = Mendelian randomization, OR = odds ratio.

To establish the directionality of the observed associations, a reverse MR strategy was used to explore the effects of reverse causality in the analysis of depression and physical health problems, especially thyroid nodule and irregular menstruation. The results from this reverse MR, as depicted in Figure 6 and thoroughly outlined in Table 3, confirmed that there was no reverse causation between depression and either thyroid nodules or irregular menstruation, where significant causal effects were previously established, each showing an IVW_*P* value of 0.64. The absence of a reverse causal relationship suggests that the genetic determinants of thyroid nodules and irregular menstruation do not precipitate depression. Conversely, the SNPs associated with depression exert a 1-way influence, modulating biological processes pertinent to depression. These findings bolster the notion that depression could potentially drive the development of thyroid nodules and menstrual irregularities, rather than the other way around.

**Table 3:** Reverse Mendelian randomization results.

Outcome	Exposure	Method	nSNP	*P* value	OR 95% CI
	Thyroid nodule	IVW	4	.25	1.012 [0.991, 1.033]
Depression	Flatulence	IVW	37	.91	0.705 [0.002, 229.292]
	Irregular menstruation	IVW	9	.06	29.791 [0.9, 986.502]
	Thyroid nodule	IVW	5	.26	1.026 [0.982, 1.071]
Anxiety	Flatulence	IVW	38	.97	0.825 [0, 16,730.477]
	Irregular menstruation	IVW	10	.10	0.045 [0.001, 1.786]

The results of the reverse Mendelian randomization analysis indicate that there is no reverse causal effect between depression and thyroid nodule (IVW_*P* value = 0.64) and between depression and irregular menstruation (IVW_*P* value = 0.64). This suggests that these SNPs influence the occurrence of thyroid nodule and irregular menstruation solely through depression. Similarly, there is no reverse causal effect for the other conditions as well.

CI = confidence interval, IVW = inverse variation weighting, OR = odds ratio, SNP = single nucleotide polymorphism.

### 4.9. Multivariable analysis

To simultaneously control for multiple confounding factors, isolate the unique effect of the exposure, and accurately determine the causal relationships between mental health and physical health outcomes, we applied multivariable analysis. The results of the analysis adjusted for the effects of anxiety and depression showed no relationship between these psychological disorders and thyroid nodules or flatulence. However, for menstrual irregularity, when anxiety is not observed, depression makes it appear (IVW_*P* value = 0.003, OR = 1.0076), indicating a supportive role carry. These findings are described in Table 4. After adjusting for depression and anxiety risk factors, multivariate analysis showed no significant psychological effect on thyroid nodules or flatulence. However, for irregular menstruation, depression still significantly promoted the condition even after accounting for anxiety. The IVW *P*-value was significant at .003, and the OR was 1.0076, showing the true and measurable impact of depression on children. The continuation of these results suggests that anxiety is a possible factor in the etiology of menstruation without anxiety. In contrast, anxiety did not have a significant effect on irregular menstruation after adjusting for the occurrence of depression.

**Table 4:** Multivariable analysis results.

Exposure	Outcome	nSNP	*P* value	OR 95% CI
Anxiety	Thyroid module	83	.419	0.839 [0.548, 1.284]
Depression		83	.315	1.288 [0.787, 2.109]
Anxiety	Flatulence	82	.410	1.000 [0.999, 1.000]
Depression		82	.320	1.000 [0.999, 1.000]
Anxiety	Irregular menstruation	82	.917	1.000 [0.996, 1.005]
Depression		82	.003	1.008 [1.003, 1.013]

When the *P*-value is less than .05, the result is considered significant; otherwise, there is no causal effect.

CI = confidence interval, OR = odds ratio, SNP = single nucleotide polymorphism.

## 5. Discussion

Recent studies have shown that depression is not only a profound psychological burden but also has a significant impact on physical health.^[24]^ It is linked to chronic ailments, such as cardiovascular disease and diabetes, as well as endocrine disorders, such as thyroid dysfunction.^[25–27]^ For instance, recent studies suggest that chronic depression can lead to dysregulation of the hypothalamic-pituitary-thyroid (HPT) axis, characterized by alterations in the secretion of thyrotropin-releasing hormone from the hypothalamus and thyroid-stimulating hormone from the pituitary gland, ultimately affecting thyroid hormone levels and potentially increasing the risk of thyroid-related diseases.^[28]^ Additionally, prolonged HPT axis dysregulation may also influence the local thyroid tissue microenvironment, including alterations in cytokine secretion and oxidative stress levels, which contribute to the initiation and progression of thyroid nodules.^[29]^ Likewise, anxiety disorders have been shown to correlate with a range of physical ailments potentially caused by a chronic anxiety response that can alter gastrointestinal, cardiovascular, and hormonal functions.^[30,31]^ Clinical studies have reported that anxiety contributes to, or exacerbates, physical ailments. For example, studies by Lim et al^[32]^ and Olafiranye et al^[33]^showed a significant association between anxiety and conditions such as irritable bowel syndrome and an increased risk of coronary heart disease. However, these studies frequently face challenges related to confounding variables, a limitation that MR methodologies are specifically designed to address. In this study, we aimed to utilize MR to disentangle the causal relationship between depression/anxiety and physical conditions, and to clarify whether associations observed in epidemiological studies are driven by confounding factors or represent genuine causal effects.

The formation of thyroid nodules is thought to be affected by psychological factors, with depression having a more significant effect than anxiety.^[34]^ A potential biological pathway involves alterations in thyroid hormone metabolism, which may thereby contribute to the development of thyroid nodules.^[35,36]^ In this study, it is noted that the OR value for the association between depression and thyroid nodules is noteworthy, which preliminarily indicates a causal relationship between depression and thyroid nodules. Meanwhile, the association between anxiety, depression, and thyroid dysfunction highlights the need for careful monitoring of thyroid health in individuals with depression.

As elucidated by prior researchers, gastrointestinal manifestations such as flatulence frequently arise from behavioral modifications linked to depression, which lend substantial support to the hypothesis that psychological factors play a substantial role in the pathogenesis of flatulence.^[37,38]^ Based on this, recognizing these etiological factors enables healthcare providers to adopt targeted interventions to effectively alleviate gastrointestinal symptoms.^[39]^ However, in this study, our MR analysis failed to identify any direct causal associations between depression, anxiety, and flatulence. Regarding this discrepancy, we have conducted several analyses as follows. Firstly, our study utilized a MR approach to evaluate causality, a methodological divergence from previous observational studies that likely explains the differences in results. Specifically, while residual confounding may persist in traditional observational research, our genetic-based approach was designed to minimize such biases. Additionally, the associations identified in prior studies might reflect intricate bidirectional relationships; for instance, gastrointestinal symptoms could potentially exacerbate depression, creating a feedback loop that observational methods may misinterpret as a unidirectional causal link. Finally, disparities in the specific phenotypes under investigation and the characteristics of the study populations across different research efforts can contribute to the varying outcomes observed.

Moreover, irregular menstruation was influenced by disorders of the HPT axis that regulate hormone production.^[40]^ In this study, we discovered a link between depression and irregular menstruation. This connection may be mediated by depression-induced disruption of the hypothalamic-pituitary-gonadal axis, which in turn affects the production of key hormones.^[41,42]^ In addition, this hormonal imbalance can be directly caused by changes in the release of gonadotropin-releasing hormone, which is often caused by anxiety and depression. Therefore, clinicians should consider these findings when treating depression in women. Notably, due to the nonsignificant outcome of the reverse MR analysis and the extremely wide CI, these results cannot be reliably interpreted to indicate a reverse causal relationship. Additionally, although the observed association between depression and irregular menstruation is characterized by a relatively small effect size, its implications at the population level should not be overlooked. Our findings underscore the necessity for more comprehensive investigations into the intricate relationship between depression and reproductive health, thereby facilitating the development of integrated strategies for managing both mental well-being and menstrual health in affected individuals. Further research is needed to test these relationships in different populations and to integrate genetic data with environmental and lifestyle factors to understand their interactions.^[43]^

Methodologically, the use of extensive datasets and multiple MR techniques has significantly enhanced the rigor of the analytical results. Including extensive data from reputable genetic databases, such as PGC and FinnGen, increases the statistical power of analyses and ensures that findings come from diverse and representative samples. These databases are noted for their extensive collection of genetic data, which has been carefully collected and curated to ensure high data quality and reliability for genetic research.^[44,45]^ Moreover, employing big data is important in genetic research because it helps to achieve the necessary power to identify and interpret genetic relationships. Using a small sample size reduces random errors and improves the accuracy of predictions, thereby improving the reliability of the results. The PGC and FinnGen databases contain genetic information that helps to explore the genetics of mental disorders and their effects on physical health.^[46,47]^ In this study, a significant decrease in the number of usable SNPs for anxiety compared to depression underscores the varying genetic influences and stringent data quality controls imposed, ensuring that only the most reliable genetic instruments are used for subsequent causal inference analyses. For example, individuals with increased anxiety may engage in behaviors such as smoking, poor diet, and inconsistent sleep patterns, all of which can lead to physical health problems.^[48,49]^ Physicians must develop treatment strategies that include both mental and physical health, with emphasis on lifestyle to improve patient outcomes.^[50,51]^ In addition, incorporating data from different cultures and investigating the involvement of mediators such as anxiety processes can provide deeper insights into the effects of indirect anxiety on the body’s health through behavior or the environment.^[52,53]^

In addition to leveraging large datasets, multiple MR methods were employed to guarantee reliable and unbiased results. The use of methods such as IVW and MR-Egger is particularly important in solving the problem of pleiotropy, where genetic variants influence many traits and potentially confound MR analyses. In detail, IVW is a standard method in MR analyses that provides a combined assessment of causal effects by weighting individual genetic associations according to their inverse variance, thereby maximizing the use of available data.^[54]^ By contrast, MR-Egger introduces an intercept term to the regression model, which tests for and, if needed, adjusts for pleiotropic effects. This method is important to ensure that the MR estimates are not biased owing to pleiotropic genetic variants, making the causal inference more reliable.^[55,56]^ Moreover, employing both IVW and MR-Egger allows for cross-validation of the results, where consistency across these methods strengthens the confidence in the causal estimates reported. The potential for pleiotropy and other biases in genetic studies necessitates careful methodological consideration. Through a combination of different MR techniques, our study not only assessed but also adjusted for these biases, thereby enhancing the validity of the identified causal relationships.^[57,58]^ In this study, for irregular menstruation, the MR-Egger intercept test’s borderline *P*-value hints at potential pleiotropy, yet demands careful interpretation. Our comprehensive sensitivity analyses, particularly IVW heterogeneity tests showing no significant SNP-level heterogeneity in both significant and nonsignificant associations, underpin the robustness of our main findings. Moreover, by testing multiple datasets of depression, we found that the current SNP set generated the most significant results; excluding SNPs would lead to nonsignificant outcomes, validating our decision to retain this set for reliable causal inference. Although the *P*-value obtained from the MR-Egger intercept test resides in a marginally significant range, converging lines of evidence, including stable effect estimates from leave-one-out sensitivity analyses and visual inspection of a symmetric funnel plot, collectively suggest that pleiotropic effects are unlikely to substantially confound the study’s inferences. Nevertheless, future investigations employing larger sample sizes and an expanded repertoire of genetic instruments are imperative to rigorously disentangle the impact of pleiotropy and validate the present findings.

The strong methodologies of our study offer substantial benefits to clinical practice and ongoing studies. By demonstrating a more dependable causal relationship between mental health disorders and physical health conditions, enabling medical professionals to gain deeper insights into the genetic variables potentially impacting these health outcomes.This understanding can lead to more positive effects on mental and physical health. Additionally, the analytical approach employed in this research can serve as a model for future genetic association studies, especially those that explore the complex interactions between genetic, environmental, and behavioral influences. Understanding the genetic foundations that link mental health to physical ailments can yield important clinical outcomes. For instance, recognizing that depression may contribute to the onset of conditions such as thyroid nodules and irregular menstruation may prompt the adoption of more holistic treatment strategies in which treatment plans consider both mental and physical health.^[59]^ Future studies should examine these associations in different populations and other physical health effects associated with depression. In addition, research combining genetic information with environmental and lifestyle factors improves our understanding of the interaction between depression and health.^[60,61]^

Although this study has its strengths, it also has some limitations. Firstly, the external validity of the study is limited by the demographic homogeneity of the sample: most participants were of European ancestry. This genetic bias, alongside uneven subgroup sample sizes, may restrict the generalizability of diverse populations. Future research should prioritize multiethnic cohorts with balanced representation to enhance the robustness and applicability of the findings. Second, this study was limited to a specific set of physical health outcomes, restricting a comprehensive understanding of the bidirectional relationship between mood disorders and somatic health. Integrating advanced biomarker analyses with cutting edge artificial intelligence methodologies could enable a more nuanced exploration of the complex pathophysiological mechanisms underlying the comorbidity of depression, anxiety, and physical health conditions.^[62,63]^ This approach would not only clarify the biological pathways involved but also facilitate the development of more targeted preventive and therapeutic strategies.

## Author contributions

**Conceptualization:** Zheng Bi, Zhaohui Fang.

**Data curation:** Lujie Wang.

**Funding acquisition:** Zhaohui Fang.

**Software:** Jinju Li.

**Visualization:** Jiawen Jing.

**Writing – original draft:** Zheng Bi.

**Writing – review & editing:** Zhaohui Fang.

## Supplementary Material


